# Making the EHR Work for You—Modifications of an Electronic Health Record System to Improve Tracking and Management of Patients Receiving Outpatient Parenteral Antibiotic Therapy

**DOI:** 10.1093/ofid/ofae005

**Published:** 2024-01-12

**Authors:** Sonal S Munsiff, Colleen Burgoyne, Erica Dobson, Alexandra Yamshchikov

**Affiliations:** Division of Infectious Diseases, University of Rochester School of Medicine and Dentistry, Rochester, New York, USA; Division of Infectious Diseases, University of Rochester School of Medicine and Dentistry, Rochester, New York, USA; Department of Pharmacy, University of Rochester School of Medicine and Dentistry, Rochester, New York, USA; Division of Infectious Diseases, University of Rochester School of Medicine and Dentistry, Rochester, New York, USA

**Keywords:** EHR, electronic medical record, OPAT, outpatient parenteral antibiotic therapy, quality improvement

## Abstract

**Background:**

Managing the complex needs of outpatient parenteral antibiotic therapy (OPAT) patients is challenging and time-consuming. We describe development of multimodal interventions to facilitate patient management within an Epic® (Epic Systems Corporation)-based electronic health record (EHR) platform.

**Methods:**

During 2016–2018, a multidisciplinary team created several modifications in our local EHR to improve gaps in OPAT care, including shared note templates, shared patient lists, automatically triggered notifications, and comprehensive order sets. A SmartForm was created, allowing collection of discrete, self-contained extractable data about each OPAT episode. We reviewed OPAT episodes from January 2019 through December 2022.

**Results:**

The multimodal EHR interventions culminated in the creation of a patient report, the “OPAT Monitoring View” collating OPAT-relevant data from multiple sections of the chart onto 1 screen display. This view is accessible both within the patient chart and from multiple list-based, in-basket, and snapshot-anchored preview functions in the EHR. Implementation of the EHR bundle facilitated management of 3402 OPAT episodes from 2019 to 2022 (850 episodes/year), about 50% higher than anticipated based on 540 OPAT courses in 2016. The OPAT EHR bundle allowed efficient (<3 hours) multidisciplinary rounds for management of 130–145 patients each week, streamlining of care transitions, and increasing staff satisfaction.

**Conclusions:**

Bundled multimodal modifications to the local EHR increased patient care efficiency and staff satisfaction and facilitated data collection to support a large OPAT program. These modifications apply commonly available EHR functionalities to OPAT care and could be adapted to other settings with different EHR platforms.

## Teaser

Successful OPAT care requires coordination with multiple agencies in addition to the patient, family and infectious diseases clinical team. The EHR is often a barrier to the complex monitoring required. Modifications to the EHR allowed for improved safety, communication and efficiency.

Outpatient parenteral antibiotic therapy (OPAT) is a safe, efficacious, and cost-effective modality for providing essential treatment outside the hospital setting for serious infections [1–3]. OPAT allows patients to return to regular activities, with increased satisfaction and less disruption to life, and reduces risks associated with prolonged hospitalization, including acquisition of drug-resistant organisms [4–7]. Administration of OPAT can be complex and challenging, requiring supervision of prolonged antibiotic courses in various medical and nonmedical settings. A health care failure mode effect analysis (HFMEA) to delineate OPAT processes and associated vulnerabilities found that 6 main OPAT processes and 67 subprocesses included potential for communication failures between the OPAT team, the patient, and multiple external agencies facilitating OPAT [8]. These findings highlight the need for developing efficient and robust communication systems.

Updated OPAT guidelines from the Infectious Diseases Society of America (IDSA) reaffirm the central role of Infectious Diseases (ID) physicians in ensuring the efficacy and feasibility of OPAT [3]. Mandatory ID consultation for patients at the start of an OPAT episode improves stewardship of broad-spectrum or otherwise unnecessary antibiotics, decreases utilization of intravascular access devices (IVADs), and promotes cost-savings while maintaining equivalent clinical outcomes [9, 10]. The IDSA guidelines further emphasize the need for close and extensive patient monitoring during OPAT, with a focus on timely and available therapeutic drug monitoring bloodwork, assurance of safety and integrity of IVADs, and monitoring for toxicities and side effects [3]. Appropriately collected and readily available laboratory results are known to lessen readmission rates for OPAT patients and capture serious side effects [11, 12]. However, ensuring that laboratory results for patients in a variety of settings and over a wide geographical area are obtained and reviewed by a responsible clinician in a timely manner presents a significant practical challenge and requires significant coordination between the OPAT program and different home care agencies, skilled nursing facilities, dialysis centers, and laboratories. These challenges of communication and laboratory monitoring are near universal for different OPAT programs. A recent Emerging Infections Network survey of ID specialists with experience in OPAT highlighted lack of administrative, information technology, and financial support as common barriers to developing effective OPAT programs [13]. The survey also cited specific gaps in consistent access to laboratory results and communication with other OPAT team members despite widespread adoption of EHR systems for patient care in the United States [14].

Unfortunately, rather than augmenting human cognition in these tasks, the EHR often creates barriers. The vast amount of data available in the EHR challenges clinicians to find and highlight the information that is most relevant for OPAT monitoring, leading to difficulties in creating a meaningful clinical narrative and contributing to increased cognitive load for clinicians [15, 16]. Published guidelines for balancing patient safety and program efficiency within high-volume OPAT programs are lacking, as are specific recommendations for leveraging the EHR toward the same goals. Modifications by frontline OPAT clinicians can be instrumental in transforming the EHR to facilitate patient monitoring and communication.

Managing the complex needs of OPAT patients has been challenging and time-consuming at our organization. Despite multiple systems to identify patients discharged on OPAT, gaps in capture and appropriate monitoring have continued, alongside escalating provider dissatisfaction with growing census and extensive hours of OPAT oversight. Several cases highlighted the need for realignment of program processes and tools for clear communication, therapeutic drug monitoring, and IVAD management. Delayed monitoring of drug levels due to missed orders and lack of discharge notification caused instances of avoidable renal toxicity, and failure to ensure IVAD removal after completion of therapy resulted in IVAD-associated bacteremias. Morbidity and potential mortality of such cases prompted a root cause analysis of EHR-based processes leading to modifications for easier patient identification and management. We describe the development of a bundled multimodal intervention to facilitate OPAT management within an Epic® (Epic Systems Corporation)-based EHR.

## METHODS

Two of the authors (S.S.M. and E.D.) conducted a gap analysis to identify ways to improve the capture and care of OPAT patients through EHR modifications at the University of Rochester Medical Center (URMC). Our goal was to work within the EHR itself, where all clinical work and patient monitoring are already contained, rather than utilizing an external database to track OPAT patients, as has been done by other programs [17, 18]. In 2016, several years before release of the Epic® OPAT foundation workflows [19], a multidisciplinary team comprised of the ID physician, ID pharmacist, and information technology analysts of URMC was created for optimizing the EHR to meet the needs of the program. The multimodal OPAT EHR intervention bundle included (1) shared OPAT patient lists, (2) an Infectious Diseases consulting physician “ID Plan of Care ” (POC) note template, (3) a shared and traceable order set for home antibiotic infusions and supplies, inclusive of requisite therapeutic drug monitoring orders and OPAT-specific patient discharge instructions, (4) a SmartForm-enabled OPAT Registered Nurse (RN) sign-on and sign-off note template, (5) an automatically triggered communication letter template for home care agencies, and (6) a custom OPAT monitoring snapshot report (“OPAT Monitoring View”). Details of these modifications are discussed in the following sections. A clinical policy requiring mandatory ID consultation for patients planned for OPAT was implemented from November 2017, ensuring that treatment plans were reviewed and approved by ID team before discharge.

Following completion of the EHR build, extensive provider and stakeholder education was timed to complement the launch of the improved OPAT processes. The SmartForm-enabled infrastructure allowed tracking of program expansion over time, assessment of clinical and demographic trends within the OPAT population, and evaluation of workload analytics related to support of OPAT program functions. Data entered into the SmartForm were extracted on all patients with an OPAT start date between January 1, 2019, and December 31, 2022, and are presented below.

The study was a quality improvement project and did not require review and approval by the local ethics review committee or patient consent.

## RESULTS

**Figure 1.**
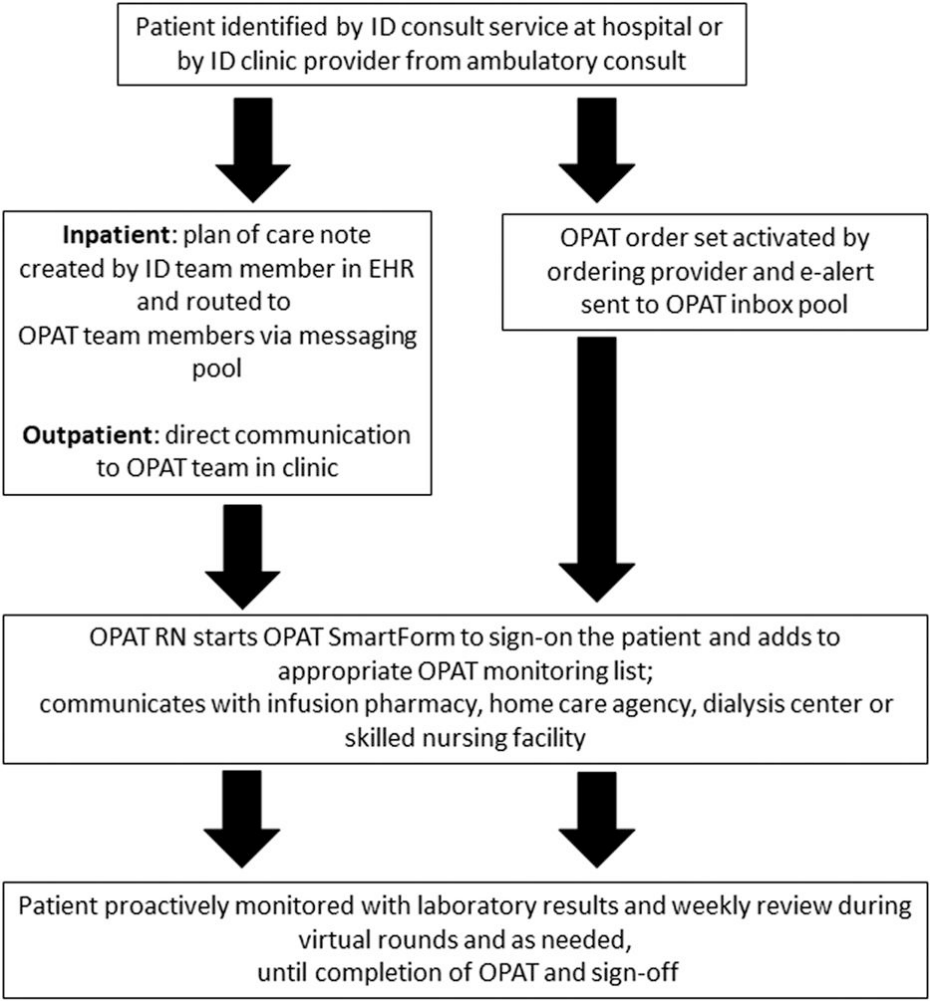
Overview of the workflow for management of patients referred for outpatient parenteral antibiotic therapy. Abbreviations: ID, Infectious Diseases; OPAT, outpatient parenteral antibiotic therapy.

The EHR modification bundle, along with addition of dedicated staff members to the OPAT program, allowed for the development of a standardized workflow aimed to optimize efficiency and minimize errors. The workflow presented in Figure 1 was established in 2016. The individual elements of the workflow are deployed around shared patient lists, accessible to all ID faculty and clinic staff, categorizing patients according to type of therapy and allowing prioritization of rounds and interventions. The High Risk/Therapeutic Drug Monitoring (TDM) list contains patients receiving oral or IV agents with prominent toxicity profiles, such as daptomycin, linezolid, and trimethoprim/sulfamethoxazole, and antibiotics that require pharmacokinetic drug level monitoring, such as vancomycin, azole antifungals, and aminoglycosides. A second list includes patients receiving IV antibiotics with lower risk of toxicity, mostly IV beta-lactams. Two additional lists capture unique patient populations shared across subspecialty providers, 1 for ventricular assist device–related infections and another for prolonged complex antibiotic therapy for nontuberculous mycobacterial infections.

To facilitate care transitions for OPAT patients following discharge, a standardized POC note template was created as a handoff and patient capture tool, outlining history of present illness, microbiology and antibiotic plan, antibiotic start and stop dates, necessary laboratory monitoring, ID clinic follow-up appointments, and unresolved microbiology or imaging results requiring outpatient follow-up (Supplementary Figure 1). The note is created by the inpatient ID team during sign-off, once antibiotic and management plans are finalized, and is manually routed to a shared OPAT In-Basket pool, which includes the OPAT RN and Advanced Practice Provider (APP). Since actual discharge dates can change after ID service sign-off, inpatient teams complete discharge arrangements and are responsible for discharge orders for outpatient IV and oral antibiotics, laboratory tests, and IVAD care supplies.

**Figure 2.**
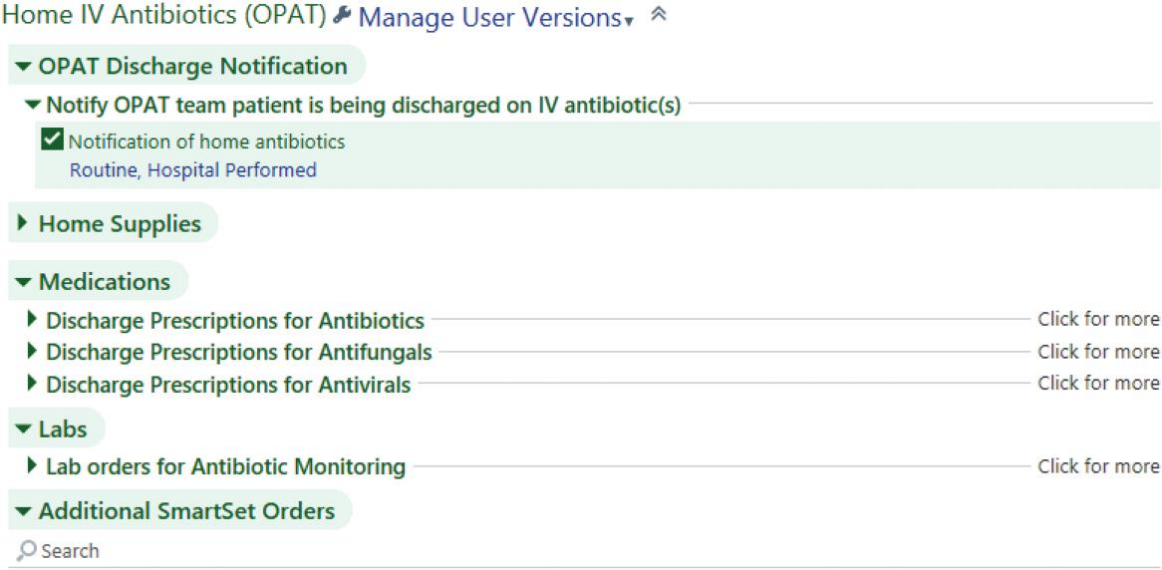
Screenshot of “home IV antibiotic” order set. The home supplies section has preselected options for IV access device care supplies, such as dressing kits and flushes. The medication section has drop-down menus of orders for all of the antibiotic, antifungal, and antiviral medications used regularly by our institution. Within these orders, there are buttons for the appropriate dosing options. The lab section has a drop-down menu for preselected standing orders for weekly (or as-needed) and therapeutic drug monitoring labs. The last section of the order set has preselected patient discharge instructions to automatically populate into the after-visit summary on how to care for their IV access device. © 2022 Epic Systems Corporation. Abbreviations: IV, intravenous; OPAT, outpatient parenteral antibiotic therapy.

An OPAT order set was built to facilitate accurate electronic prescribing of antimicrobials, supplies for home infusions, pertinent laboratory tests, and auto-populated patient instructions for IVAD care for the after-visit summary (Figure 2). The order set is configured to automatically message the OPAT In-Basket pool when deployed, a built-in redundancy for identification and capture of patients. By combining all necessary orders and discharge instructions in 1 tool, similar to pre-EHR carbon-copy paper order sets, the electronic OPAT order set was attractive and time-saving for inpatient teams discharging patients and ID providers placing OPAT orders in the outpatient setting.

**Figure 3.**
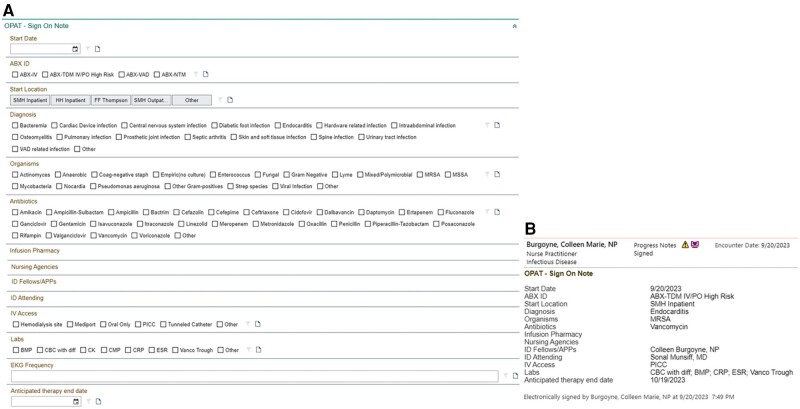
Screenshot of data fields collected for each new episode of outpatient parenteral antimicrobial therapy in the OPAT SmartForm in the EHR. *A*, Common diagnoses, organisms, antimicrobials, and other data are already populated as buttons for efficient completion of the form, rather than as drop-down menus. *B*, A note is automatically generated from the SmartForm data, and can be edited if needed, and placed in the progress note section for easy access by all staff involved in patient care. © 2022 Epic Systems Corporation. Abbreviations: EHR, electronic health record; OPAT, outpatient parenteral antibiotic therapy.

Receipt of the POC note or deployment of the OPAT order set triggers the OPAT workflow, prompting the team to add patients to shared patient lists for monitoring, investigate whether mandatory ID consultation has occurred, and begin the sign-on process. The OPAT team starts an OPAT SmartForm using information from the ID POC note (Figure 3*A*). The SmartForm is comprised of sign-on, follow-up, and conclusion of therapy sections. The sign-on section captures antibiotic regimen and clinical syndrome, infusion pharmacy, home health nursing agency, skilled nursing facility or hemodialysis site administering the course, blood work orders, and type of IVAD in place for easy retrieval of relevant information, to help efficiently supervise the particular OPAT episode. The sign-on SmartForm (1) auto-populates a progress note template that can be edited and is added to the patient's chart (Figure 3*B*) and (2) creates a letter to the home care agency that can be faxed via the EMR, clearly identifying the OPAT team as taking responsibility for the prescribed course, and provides specific information for required blood work (Supplementary Figure 2). The follow-up section of the OPAT SmartForm documents TDM data, incurred adverse drug events, IVAD problems, missing results, follow-up imaging requests, and any changes to treatment based on final microbiology results and financial issues. The conclusion of therapy section documents stoppage of antibiotics, confirmation of IVAD removal, transition to oral antibiotics if needed, and any ongoing blood work or follow-up imaging needs and auto-populates a conclusion of therapy progress note (Supplementary Figure 3*A* and *B*).

**Figure 4.**
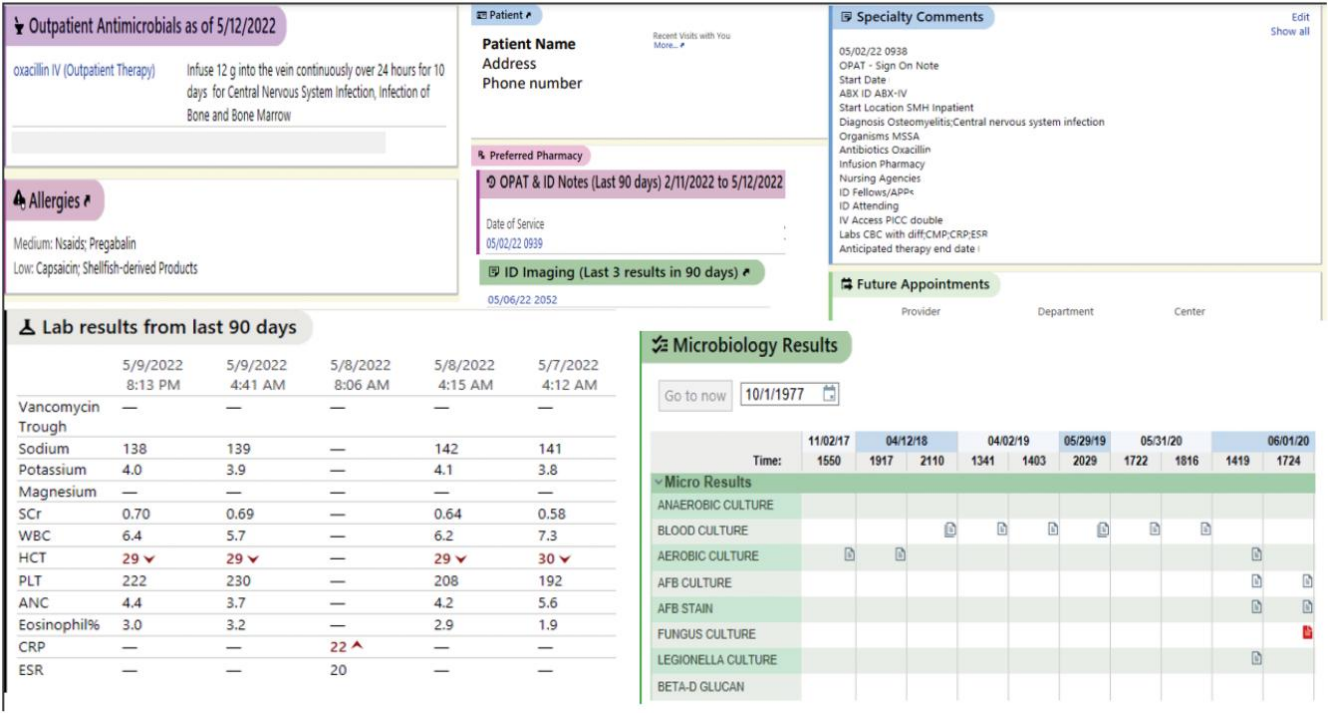
“OPAT Monitoring View” snapshot report. This view shows relevant data collated from multiple sections of the patient chart onto 1 screen display, viewable without opening the full chart. © 2022 Epic Systems Corporation. Abbreviation: OPAT, outpatient parenteral antibiotic therapy.

The bundled EHR interventions culminated in a new snapshot report, the “OPAT Monitoring View” (Figure 4), designed to increase efficiency of reviewing OPAT patients. The view projects OPAT-relevant data from multiple EHR sections onto a single screen, viewable both within the chart and within the preview function of patient lists and In-Basket messages. Information entered in the SmartForm during sign-on, upcoming appointments, blood work results from past 90 days, recent microbiology, imaging results, and ID provider and ID clinic staff notes can all be previewed and accessed from this screen. The OPAT team selected laboratory results relevant to antimicrobial monitoring, such as drug levels, electrolytes, kidney function, liver function, and complete blood counts, to be displayed. Outside laboratory results are manually entered upon receipt, automatically populating the “OPAT Monitoring View.” This report view markedly decreases propounding and rounding time, allowing OPAT team members to quickly assess whether each patient is meeting monitoring, therapeutic, and follow-up benchmarks and to troubleshoot issues in real time. The “OPAT Monitoring View” can also be used by on-call ID providers for efficient triage.

The OPAT workflow is centered on weekly virtual rounds where all patients on the OPAT lists are reviewed with the pharmacist, APP, ID physician, and OPAT RN on Wednesday mornings. The program strives to obtain TDM and other bloodwork on Mondays, so that either results are available for review by Wednesday rounds or a decision can be made to re-time collection. Rounds start with the High Risk/TDM list to ensure that patients receiving high-toxicity antimicrobials are reviewed first. Virtual rounds are led by the OPAT APP, who reviews the patient’s name, infection receiving treatment with OPAT, antibiotic regimen and planned stop date, whether a timely follow-up ID appointment was scheduled, and weekly laboratory results for abnormalities, followed by a brief exploration of barriers precluding OPAT stoppage as planned (eg, outstanding imaging results, unfavorable trends in inflammatory markers, poorly timed follow-up appointment). Abnormal results are either addressed by team consensus or deferred for broader discussion via In-Basket messages to primary ID attendings and other clinicians, such as primary care providers. On average, 130–145 patients are reviewed during OPAT rounds lasting 2.5–3.5 hours. Patients remain on OPAT lists until the team has received confirmation that antibiotics can be stopped, IVAD has been removed or signed over to another team, and an OPAT sign off note is charted.

### Characteristics of OPAT Episodes From 2019 to 2022

The OPAT bundle was fully implemented by October 2018. From January 2019 to December 2022, 3402 OPAT episodes were initiated, including 330 (10%) courses of complex outpatient antibiotic therapy (COpAT) with oral agents (Table 1). The annual average was 850 episodes per year, for an overall >50% increase in volume from 2016, when 540 OPAT episodes were monitored. The weekly census of active patients was 130–145. Antimicrobial regimens were comprised of 69 different agents. Vancomycin was the most common agent utilized (22.7% of episodes) (Table 1). The most common infections were bone/joint infections or bacteremia/endocarditis. Coordination of care was required for 513 episodes with 60 skilled nursing facilities, and for 225 episodes with 30 hemodialysis sites. For home-based episodes, we interacted with 39 home care agencies and 16 infusion pharmacies to coordinate antibiotic doses, delivery of drugs and infusion supplies, central line care, and blood draws. Anecdotally, physician satisfaction has improved as a result of improved efficiency and safety of OPAT care and the simplified process for ordering antibiotics, blood tests, and IVAD care supplies.

**Table 1.** Clinical Characteristics of OPAT Episodes From 2019 to 2022

OPAT episodes (average/y)	3402 (850.5/y)
Median OPAT duration (IQR), d	30 (24)
OPAT start locations, No. (%)	
Hospital A	2106 (61.9)
Hospital B	700 (20.6)
Hospital C	128 (3.8)
Other hospitals	54 (1.6)
ID outpatient clinic	414 (12.2)
Clinical diagnoses (>1 diagnosis per episode possible), No. (%)	
Bone and joint infections	1467 (43.12)
Bacteremia/endocarditis	1177 (34.6)
Skin and soft tissue infections	268 (7.88)
Intra-abdominal infections	248 (7.29)
Cardiac device (including VAD) infections	186 (5.47)
Pulmonary infections	164 (4.82)
Central nervous system infections	131 (3.85)
Urinary tract infections	119 (3.5)
Other sites of infection	49 (1.4)
Home-based OPAT episodes, No. (%)	2664 (78.3)
Patients initiated OPAT in outpatient setting	247
Unique home nursing agencies involved	39
Unique infusion pharmacies involved	16
Unique infusion centers involved	10
Skilled nursing facility–based OPAT episodes	513 (15)
Unique skilled nursing facilities involved	60
Hemodialysis center–based OPAT episodes	225 (7)
Unique hemodialysis sites involved	30
Episodes with oral antibiotics only	330 (10)
Unique antimicrobial agents managed (including IV and oral agents)	69
Top 5 antimicrobial agents, No. (% of regimens with agent)	
Vancomycin	772 (22.7)
Ceftriaxone	688 (20.2)
Cefazolin	670 (19.7)
Cefepime	223 (6.6)
Ertapenem	189 (5.6)
Regimens with >1 antimicrobial agent	875 (25.7)
Regimens with >1 IV antimicrobial agent	261 (7.7)

Abbreviations: IQR, interquartile range; IV, intravenous; OPAT, outpatient parenteral antimicrobial therapy; VAD, ventricular assist device.

## DISCUSSION

Bundled multimodal modifications made to the local EHR at our institution, in conjunction with other programmatic and staff changes, have allowed us to support a >50% increase in the volume of OPAT episodes since this program was implemented. The cognitive load and time required for clinicians to find relevant information within the EHR are known to impact clinician burnout and patient care outcomes, partly because most EHRs remain focused on documents instead of data, a relic from the paper chart [15, 20]. Shifting the focus toward data would enable clinicians to access relevant information more efficiently and in a more comprehensive way. The EHR also creates siloed and opaque workflows and presents major barriers to team-based care, an approach known to advance OPAT patient safety and reduce clinician burnout [21]. We report a bundle of EHR modifications such as the “OPAT Monitoring View” and other EHR tools to create a more data-focused snapshot to optimize clinician time and patient monitoring processes for OPAT.

Combined, these modifications create a shared network of data, communication, and a set of shared knowledge about each patient, facilitating team-based care. Allowing multiple team members to share tools for monitoring facilitates coordination and communication and ensures that appropriate OPAT benchmarks such as mandatory ID consultation, monitoring parameters, and confirmatory steps such as IVAD removal and timely follow-up are met.

In addition to improving the efficiency of patient care and clinician satisfaction, these tools have enabled us to describe the volume and characteristics of OPAT patients in our program. Understanding and tracking the number of different home care agencies, infusion pharmacies, and skilled nursing facilities have elucidated the complexity of care required. Relationships between multiple stakeholder organizations demonstrate that OPAT is more complex than merely coordination between patients, families, and the clinical team. The ability to extract EHR encounter data for each OPAT episode has also allowed us to quantify the workload associated with OPAT, providing justification for additional staff to accommodate increased volume [22, 23].

Ongoing challenges include efficiently obtaining external laboratory results, staff time for manual entry of external results, continued reliance on phone and fax communications with multiple external agencies due to lack of electronic EHR-based messaging, and lack of staff to conduct quality improvement activities relative to patient outcome data and adverse event rates.

Communication failures are known to be at the root of many OPAT vulnerabilities [8]. Optimization of shared patient lists to include calendar-enabled “reminder” functions can help identify incomplete tasks, decrease siloed workloads, and facilitate sharing of task ownership between OPAT team members, but does little to decrease reliance on faxed or phone communications with external agencies. Additional solutions requiring further automation can be considered, such as auto-routing of POC notes and other OPAT documentation, implementation of eFax (electronic fax) folders to save faxed documents automatically as Portable Document Format (.PDF) files to decrease lost paper faxes, streamlining laboratory retrieval, review of data for clinicians, and interfacility communication.

The most pronounced barrier to the efficiency of OPAT management remains availability of monitoring blood work results. A point prevalence survey during a typical Wednesday virtual rounds identified 113 patients who required blood work that week. Of the 84 (74%) planned for laboratory processing in the URMC network, only 64 (77%) had completed results available in the EHR for weekly rounds. Among 29 patients for whom non-URMC facilities processed blood work, 16 (55%) had completed results for rounds (received via fax and manually entered by OPAT staff, or via EHR electronic data sharing agreements), 4 (14%) were received by fax but not yet entered, and 9 (31%) were otherwise not drawn or unavailable. Overall, 81 (72%) of planned 113 weekly OPAT blood samples were collected and available by Wednesday morning rounds. These results remain consistent over several weeks of repeated surveillance. Availability of laboratory results has improved, based on anecdotal experience of OPAT providers spanning the study period. The EHR itself has evolved, with result-sharing functionalities allowing improved access to organizations using similar EHR platforms. Additionally, the URMC hospital system has increased its geographic footprint over the study period, acquiring additional laboratory testing sites, local health centers, and nursing facilities, and integrating them in our EHR. Additional solutions to improve actionable availability of blood work can include procuring access to regional data-sharing collaboratives, such as the Rochester Regional Health Information Organization (RHIO) within our region, national laboratory organizations, such as Labcorp and Quest Diagnostics, and implementation of eFax folders.

Additional gap analysis related to the postdischarge period suggested ongoing errors in ordering of antimicrobials during transition from inpatient to outpatient, despite widespread adoption of the OPAT order set. We continue to refine the order set to minimize errors. A “Transitions of Care” intervention is also piloting on certain URMC patient care floors, where OPAT team members review arrangements and place the discharge orders required to execute the planned OPAT episode, rather than rely on the discharging team.

Throughout the study period, visually identifying in-patients (and therefore readmissions) has become easier due to enhanced preview functions of shared patient lists, prompting program staff to start investigating the circumstances leading to readmission during Wednesday OPAT rounds. Additional notification regarding readmissions and emergency room visits improved with implementation of the Continuity Clinic Provider (CCP) In-basket tool for ID and OPAT providers, allowing subspecialty providers to automatically receive notifications as a carbon copy (cc) to primary care provider (PCP) notifications. Adverse events and “near-misses” identified through these mechanisms, and others, are brought for discussion and root cause analysis during monthly OPAT Program Operations meetings and ID faculty meetings.

Most importantly, staffing constraints have continued to hamper evaluation of program effectiveness and systematic review of adverse events, readmissions, and IVAD-related complications. The OPAT SmartForm, created as part of the multimodal EHR intervention, includes a section for systematic capture of adverse events and readmissions. Completion of that section remains dependent on manually entering the type of event, date of onset, and degree of severity based on predefined criteria once the event is identified. Data entry tasks are not routinely perceived as impactful for day-to-day patient care and safety and are preferentially deferred during periods of high clinical workload, monitoring acuity, or during fluctuations in program staffing, leading to under-reported and inconsistent data. Additional staffing would allow us to assess the optimal frequency of lab monitoring for specific antibiotics and develop tools for identifying OPAT patients with the highest risk for central line events, adverse drug events, and hospital readmission.

Despite the creation of effective EHR-based tools to facilitate monitoring and clinical care of large volumes of OPAT patients, rapid program growth in the absence of correlative expansion of data capacity and reporting analytics can limit the program's ability to monitor quality improvement goals and demonstrate a return on financial investment within the parent organization. An OPAT program expansion to bolster non-personnel-driven reporting and analytical capacity and to allow more consistent iterative assessment of program value, readmission and length-of-stay impact, and patient safety performance is underway.

In conclusion, creating a bundle of changes in the EHR supportive of OPAT patient and staff needs required a multidisciplinary team and multiple years of development. We hope that other programs can learn from our experience and adapt their EHR more efficiently. Institutional commitment to provide dedicated staff and information technology support for necessary EHR modifications is essential for ID programs to manage OPAT patients. We are hopeful that the infrastructure generated by the bundled interventions presented in this report can advance general knowledge regarding the optimal care of OPAT patients and support OPAT programs in ensuring that care is safe and efficacious.

## Supplementary Material

ofae005_Supplementary_Data

## Supplementary Data

Supplementary materials are available at *Open Forum Infectious Diseases* online. Consisting of data provided by the authors to benefit the reader, the posted materials are not copyedited and are the sole responsibility of the authors, so questions or comments should be addressed to the corresponding author.

## References

[1] Antoniskis A, Anderson BC, Van Volkinburg EJ, Jackson JM, Gilbert DN. Feasibility of outpatient self-administration of parenteral antibiotics. West J Med 1978; 128:203–6. 636409 PMC1238051

[2] Barr DA, Seaton RA. Outpatient parenteral antimicrobial therapy (OPAT) and the general physician. Clin Med (Lond) 2013; 13:495–9. 24115709 10.7861/clinmedicine.13-5-495 PMC4953803

[3] Norris AH, Shrestha NK, Allison GM, et al 2018 Infectious Diseases Society of America Clinical Practice guideline for the management of outpatient parenteral antimicrobial therapy. Clin Infect Dis 2019; 68:e1–35. 10.1093/cid/ciy745 30423035

[4] Durojaiye OC, Bell H, Andrews D, Ntziora F, Cartwright K. Clinical efficacy, cost analysis and patient acceptability of outpatient parenteral antibiotic therapy (OPAT): a decade of Sheffield (UK) OPAT service. Int J Antimicrob Agents 2018; 51:26–32. 28673610 10.1016/j.ijantimicag.2017.03.016

[5] Madaline T, Nori P, Mowrey W, et al Bundle in the Bronx: impact of a transition-of-care outpatient parenteral antibiotic therapy bundle on all-cause 30-day hospital readmissions. Open Forum Infect Dis 2017; 4:XXX–XX. 10.1093/ofid/ofx097 PMC5570156 28852672

[6] Dimitrova M, Gilchrist M, Seaton RA. Outpatient parenteral antimicrobial therapy (OPAT) versus inpatient care in the UK: a health economic assessment for six key diagnoses. BMJ Open 2021; 11:e049733. 10.1136/bmjopen-2021-049733 PMC8479950 34588251

[7] Tice AD, Rehm SJ. Meeting the challenges of methicillin-resistant *Staphylococcus aureus* with outpatient parenteral antimicrobial therapy. Clin Infect Dis 2010; 51(Suppl 2):S171–5. 20731574 10.1086/653517

[8] Gilchrist M, Franklin BD, Patel JP. An outpatient parenteral antibiotic therapy (OPAT) map to identify risks associated with an OPAT service. J Antimicrob Chemother 2008; 62:177–83. 18408239 10.1093/jac/dkn152

[9] Conant MM, Erdman SM, Osterholzer D. Mandatory infectious diseases approval of outpatient parenteral antimicrobial therapy (OPAT): clinical and economic outcomes of averted cases. J Antimicrob Chemother 2014; 69:1695–700. 24532684 10.1093/jac/dku015

[10] Sharma R, Loomis W, Brown RB. Impact of mandatory inpatient infectious disease consultation on outpatient parenteral antibiotic therapy. Am J Med Sci 2005; 330:60–4. 16103785 10.1097/00000441-200508000-00002

[11] Huck D, Ginsberg JP, Gordon SM, Nowacki AS, Rehm SJ, Shrestha NK. Association of laboratory test result availability and rehospitalizations in an outpatient parenteral antimicrobial therapy programme. J Antimicrob Chemother 2014; 69:228–33. 23887864 10.1093/jac/dkt303

[12] Zukauckas K, Benefield RJ, Newman M, Certain L. Why bother? Lab monitoring in beta-lactam outpatient parenteral antimicrobial therapy. Antimicrob Agents Chemother 2022; 66:e0057922. 35543523 10.1128/aac.00579-22 PMC9211408

[13] Hamad Y, Lane MA, Beekmann SE, Polgreen PM, Keller SC. Perspectives of United States-based infectious diseases physicians on outpatient parenteral antimicrobial therapy practice. Open Forum Infect Dis 2019; 6:XXX–XX. 10.1093/ofid/ofz363 PMC6765349 31429872

[14] Henry J, Pylypchuk Y, Searcy T, Patel V. *Adoption of Electronic Health Record Systems Among U.S. Non-federal Acute Care Hospitals: 2008–2015*. *ONC Data Brief, No. 35*. Office of the National Coordinator for Health Information Technology; **2016**. 40127322

[15] Weir CR, Taber P, Taft T, Reese TJ, Jones B, Del Fiol G. Feeling and thinking: can theories of human motivation explain how EHR design impacts clinician burnout? J Am Med Inform Assoc 2021; 28:1042–6. 33179026 10.1093/jamia/ocaa270 PMC8068417

[16] Windle JR, Windle TA, Shamavu KY, et al Roadmap to a more useful and usable electronic health record. Cardiovasc Digit Health J 2021; 2:301–11. 35265926 10.1016/j.cvdhj.2021.09.007 PMC8890352

[17] Kaul CM, Haller M, Yang J, et al Assessment of risk factors associated with outpatient parenteral antimicrobial therapy (OPAT) complications: a retrospective cohort study. Antimicrob Steward Healthc Epidemiol 2022; 2:e183. 36406163 10.1017/ash.2022.313 PMC9672913

[18] Hemenway AN, Stewart RL. Reflections on implementation of a failure-point-focused outpatient parenteral antimicrobial therapy management program. Antimicrob Steward Healthc Epidemiol 2022; 2:e158. 36483368 10.1017/ash.2022.304 PMC9726515

[19] Epic Systems Corporation . *Outpatient Parenteral Antimicrobial Therapy Strategy Handbook*. Epic Systems Corporation; **2021**.

[20] Johnson KB, Neuss MJ, Detmer DE. Electronic health records and clinician burnout: a story of three eras. J Am Med Inform Assoc 2021; 28:967–73. 33367815 10.1093/jamia/ocaa274 PMC8068425

[21] Smith CD, Balatbat C, Corbridge S, et al Implementing optimal team-based care to reduce clinician burnout. NAM Perspectives Discussion Paper. **2018**. Available at: https://nam.edu/implementing-optimal-team-based-care-to-reduceclinician-burnout. Accessed 29 June 2023.

[22] Burgoyne C, Calisir N, Munsiff S. Quantifying the non-billable workload of outpatient parenteral antibiotic therapy (OPAT) services in a university infectious diseases (ID) clinic. Open Forum Infect Dis 2020; 7(Suppl 1):S370.

[23] Munsiff S, Burgoyne C, Goins P. A model for assessing staffing needs for an outpatient parenteral antibiotic therapy (OPAT) program. Open Forum Infect Dis 2020; 7(Suppl 1):S358–9.

