# Supplementary material for: Making the EHR Work for You—Modifications of an Electronic Health Record System to Improve Tracking and Management of Patients Receiving Outpatient Parenteral Antibiotic Therapy
Source: Open Forum Infect Dis. 2024 Jan 12;11(2):ofae005. doi: 10.1093/ofid/ofae005 (PMC10866571; doi:10.1093/ofid/ofae005)
Supplement: ofae005_Supplementary_Data [file ofae005_supplementary_data.zip › Supplementary Material OPAT EHR modifications 9-2023.docx]

**Supplementary Material**

Making the EHR work for you - Modifications of an Electronic Health Record System to Improve Tracking and Management of Patients Receiving Outpatient Parenteral Antibiotic Therapy

Sonal S. Munsiff, MD, FIDSA^1^

Colleen Burgoyne, FNP^1^

Erica Dobson, PharmD^2^

Alexandra Yamshchikov MD^1^

1. Division of Infectious Diseases, University of Rochester School of Medicine and Dentistry, Rochester, NY, United States
2. Department of Pharmacy, University of Rochester School of Medicine and Dentistry, Rochester, NY, United States

**Corresponding Author:**

Sonal S Munsiff, MD, FIDSA

Email: sonal_munsiff@urmc.rochester.edu

Open Forum Infectious Diseases

September 2023

**Supplementary Figure 1: OPAT plan of care note template**

The Infectious Diseases Plan of Care note template used for inpatients discharged to home or skilled nursing facility to continue outpatient parenteral or complex oral antibiotic therapy, summarizing the outpatient management plan. This note is manually routed to the OPAT team and outpatient ID clinician’s EHR In-Basket.

© 2022 Epic Systems Corporation

**Supplementary Figure 2: Template of letter to Home Care Agencies sent at OPAT sign-on**

© 2022 Epic Systems Corporation

**Supplementary Figure 3A and 3B: OPAT team Sign-off SmartForm and note**

3A - Screenshot of data fields collected at conclusion of each episode of Outpatient Parenteral Antimicrobial Therapy in the OPAT SmartForm in the EHR. This is completed by OPAT tema member.

3B- The SmartForm auto populates a progress note that is reviewed and can be edited, and placed in patient chart for easy access by any clinician.

© 2022 Epic Systems Corporation
