# Supplementary figures and images for: Making the EHR Work for You—Modifications of an Electronic Health Record System to Improve Tracking and Management of Patients Receiving Outpatient Parenteral Antibiotic Therapy
Source: Open Forum Infect Dis. 2024 Jan 12;11(2):ofae005. doi: 10.1093/ofid/ofae005 (PMC10866571; doi:10.1093/ofid/ofae005)

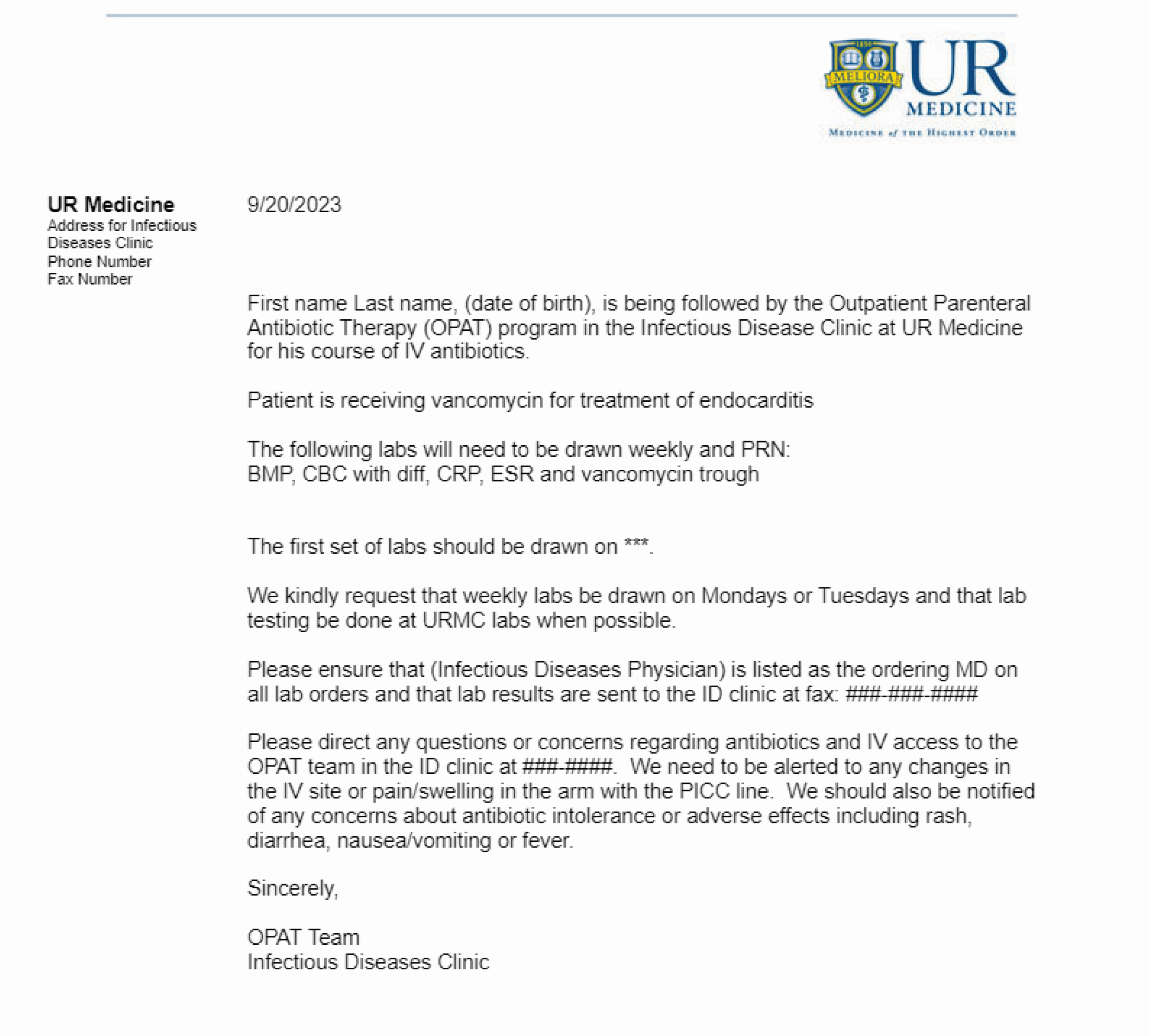

Supplement: ofae005_Supplementary_Data [file ofae005_supplementary_data.zip › Supplementary Figure 2.tif]

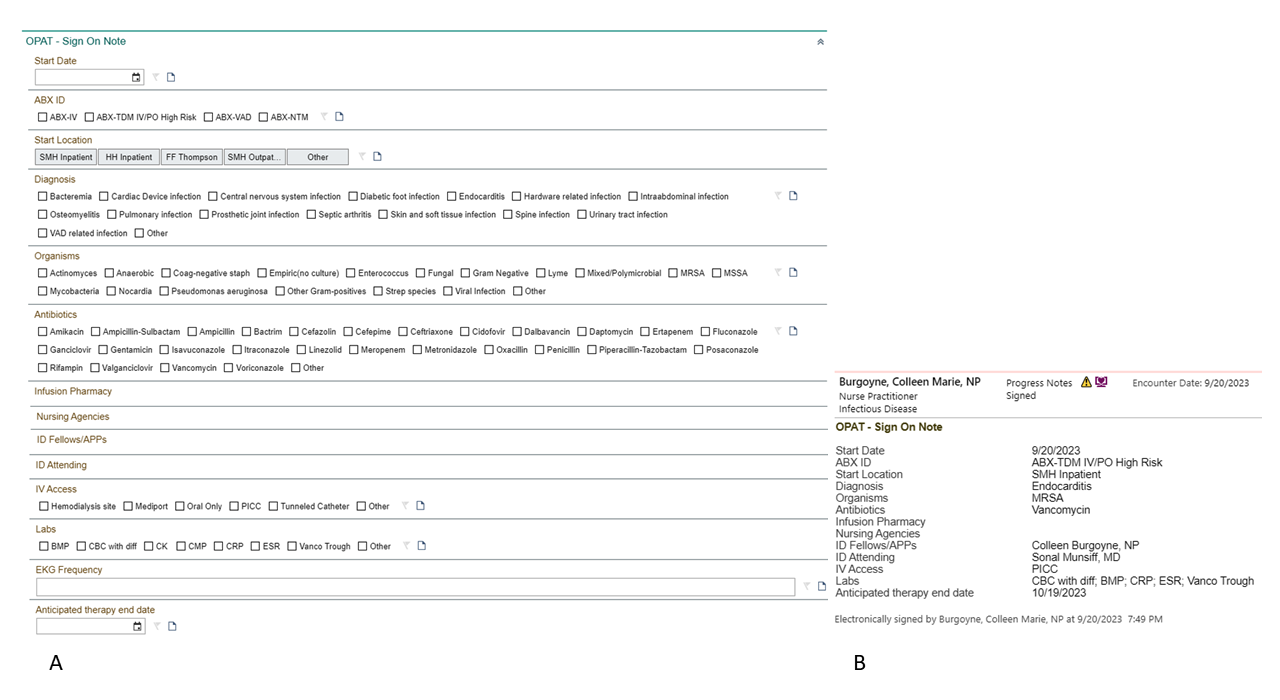

Supplement: ofae005_Supplementary_Data [file ofae005_supplementary_data.zip › Figure 3A and 3B landscape.tif]

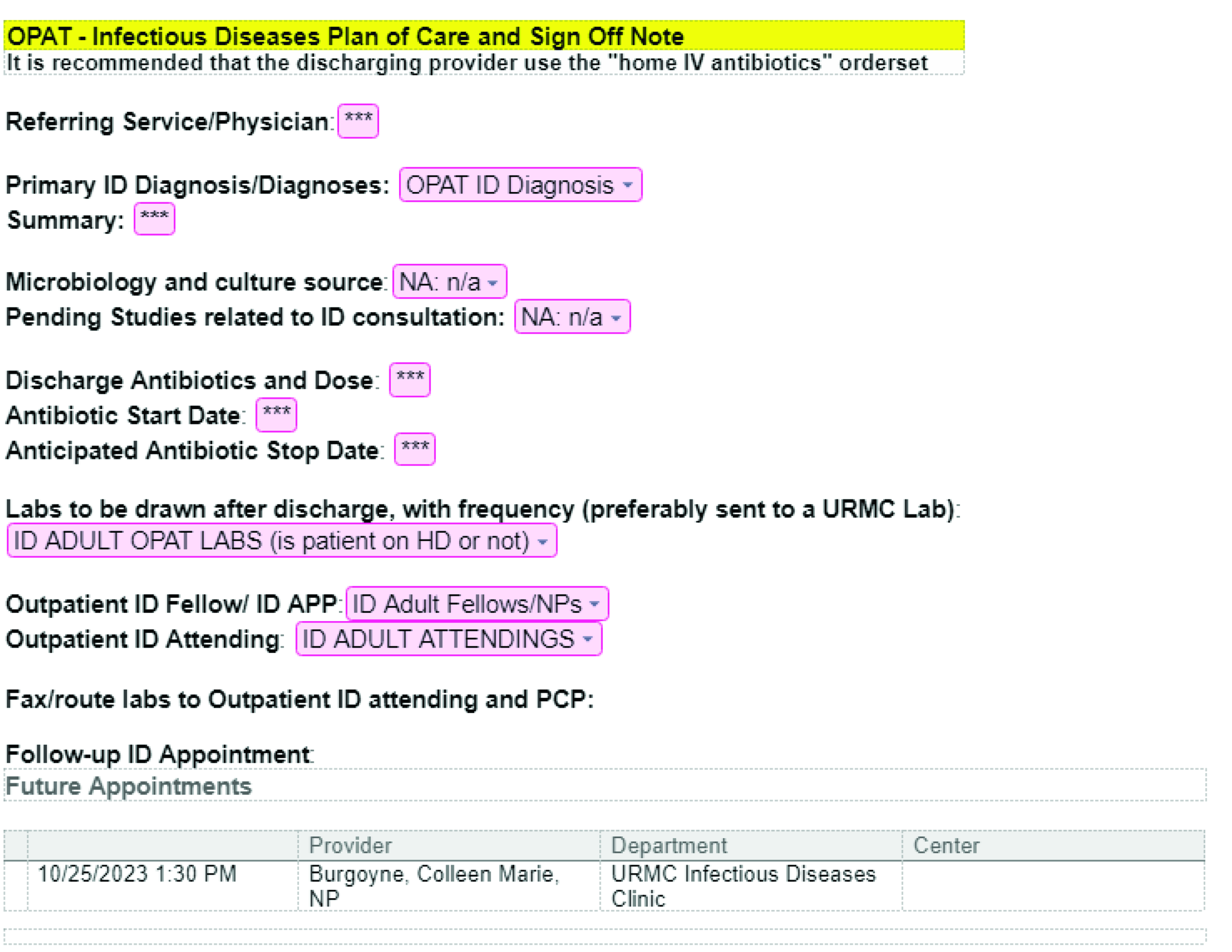

Supplement: ofae005_Supplementary_Data [file ofae005_supplementary_data.zip › Supplementary Figure 1.tif]
